# Early Duplication of a Single MHC IIB Locus Prior to the Passerine Radiations

**DOI:** 10.1371/journal.pone.0163456

**Published:** 2016-09-22

**Authors:** John A. Eimes, Sang-im Lee, Andrea K. Townsend, Piotr Jablonski, Isao Nishiumi, Yoko Satta

**Affiliations:** 1 Seoul National University, Department of Biological Sciences, Seoul, Korea; 2 Seoul National University, Institute of Advanced Machines and Design, Seoul, Korea; 3 Hamilton College, Department of Biology, Clinton, NY, United States of America; 4 National Museum of Nature and Science, Department of Zoology, Tsukuba, Japan; 5 The Graduate University for Advanced Studies, Department of Evolutionary Studies of Biosystems, Hayama, Japan; Laboratoire de Biologie du Développement de Villefranche-sur-Mer, FRANCE

## Abstract

A key characteristic of MHC genes is the persistence of allelic lineages over macroevolutionary periods, often through multiple speciation events. This phenomenon, known as trans-species polymorphism (TSP), is well documented in several major taxonomic groups, but has less frequently been observed in birds. The order Passeriformes is arguably the most successful terrestrial vertebrate order in terms of diversity of species and ecological range, but the reasons for this success remain unclear. Passerines exhibit the most highly duplicated MHC genes of any major vertebrate taxonomic group, which may generate increased immune response relative to other avian orders with fewer MHC loci. Here, we describe phylogenetic patterns of the MHC IIB in the passerine family Corvidae. Our results indicate wide-spread TSP within this family, with at least four supported MHC IIB allelic lineages that predate speciation by many millions of years. Markov chain Monte Carlo simulations indicate that divergence of these lineages occurred near the time of the divergence of the Passeriformes and other avian orders. We suggest that the current MHC diversity observed in passerines is due in part to the multiple duplication of a single MHC locus, *DAB*1, early in passerine evolution and that subsequent duplications of these paralogues have contributed to the enormous success of this order by increasing their ability to recognize and mount immune responses to novel pathogens.

## Introduction

The evolution of the vertebrate adaptive immune system is distinguished by periodic gene duplications and rearrangement, notably those genes that code for the major histocompatibility complex (MHC) [1]. These molecular rearrangements, which include both mass duplications of entire blocks of genes as well as individual genes such as those that bind and present peptides, are potentially important milestones in transitionary phases of major taxonomic groups among vertebrates [2]. Among MHC genes, the MHC IA and IIB, which code for proteins involved in peptide binding, pathogen recognition and subsequent immune response, are the most polymorphic coding loci among vertebrates, and this polymorphism is thought to be maintained by pathogen mediated balancing selection, although sexual selection and maternal-fetal interactions are also known to play an important role [3–6]. Duplications of these specific genes may influence ecological adaptation and thus have important consequences for species survival and the radiation of major taxonomic groups.

Although MHC loci are duplicated in many vertebrate lineages, there is considerable variation in the number of MHC loci expressed between divergent taxonomic groups. For example, among mammals, humans can express up to five MHC (HLA) class IIB loci while most amphibian species express just two loci [7, 8]. No terrestrial vertebrate order exhibits a higher level of MHC gene duplication than the avian order Passeriformes, specifically, the oscine suborder Passeri (songbirds), with some species expressing over 20 MHC IIB loci [9]. The Passeriformes is arguably the most diverse order of terrestrial vertebrates in terms of number of families and species as well as ecological diversity, rivaled in these attributes only by the reptilian order Squamata [10–12]. This unusually rich species diversity (69% of all birds are passerines) was the product of several taxonomic radiations among the Passeriformes beginning with the split of the passerines from Psittaciformes (parrots) *ca* 53 ma [13]. The parrot group, indeed all non-passerines with rare exceptions [14] lack highly duplicated MHC loci, and it is unclear to what extent MHC duplications influenced the great passerine radiations, but it would seem to be an unlikely coincidence.

A distinguishing characteristic of MHC genes is that alleles that code for the peptide binding region (PBR) often share more similarity between species, rather than among loci within species. This phenomenon is known as “trans-species polymorphism” (TSP), and there is strong evidence that orthologous MHC allelic lineages are maintained by selection, often over macro-evolutionary time-scales, and persist through speciation events [15]. Trans-specific clustering (TSP) of MHC loci is a potential signature of selection and the persistence of these polymorphisms among divergent lineages has been well documented in a variety of vertebrate taxa including mammals, fish and some birds [16–21]. TSP has been shown in a few recently diverged, or geographically isolated, species of passerines, with the notable exception of a recent study that showed some intermingling of MHC IIB alleles among three different passerine families [22–25]; however, a general pattern among nearly all bird species studied to date has emerged: in phylogenetic reconstructions, MHC IIB variants tend to cluster by species, rather than by locus (e.g. [26]).

There are at least two possible explanations for this observed pattern. The first possibility is that MHC IIB loci in birds is the result of ancient gene duplication, followed by episodic gene conversion that had the effect of homogenizing the MHC IIB within species or genera [27]. If this is true, there appears to be a time limit in which TSP can be detected in passerines. Indeed, a recent analysis of plant resistance genes showed that recombination (and hence, gene conversion) had a significant impact on the terminal branch lengths and the gene topologies in phylogenetic studies, which suggests that the signal of TSP in passerines can become eroded by such recombination-like processes over time [28]. Consistent with this idea, our recent study demonstrated TSP in crows and estimated a recent divergence time (< 2My) between all three species. Our results were consistent with TSP in that the combined MHC IIB sequences grouped together as if they were representative of a single species, irrespective of actual ancestry, similar to the results reported for Darwin’s finches [23].

Another explanation for the general lack of TSP in passerines is recent gene duplication, or the “birth and death” model, in which new genes continually arise via duplication with some alleles diverging further due to recombination and mutation while others become pseudogenes that may be lost or remain in the genome and contribute to increased polymorphism by recombining with functional genes [1, 29]. A variation of the birth and death model is the accordion model, in which the MHC expands and contracts over time due to selection from pathogens (Klein et al. 1993).

Here, we use a systemic approach to attempt to answer two basic questions: First, is TSP truly rare in passerines, or is the general lack of evidence for TSP in passerines due to the absence of comprehensive systematic analysis of MHC variants among the passerines? The strategy we employed to answer this first question was to focus on a single family within the passerines, the Corvidae, a basal group within the passerines, which incidentally also represents the lesser of the two great oscine radiations (the core Corvoidia), and thus may represent a simpler pattern of MHC evolution than that seen in the larger Eupasseri radiation [30]. By focusing on a single passerine family, we predicted that the signal of trans-specific retention of MHC IIB allelic lineages will be evident in phylogenetic reconstructions as opposed to typical passerine phylogenies that include inter-familial and inter-generic species, in which MHC sequences usually cluster by species (e.g. [26]).

The second question addressed by this study, is, if indeed there are ancient MHC IIB lineages within the Corvidae (as revealed by patterns of TSP), how old are these gene lineages? We used Markov chain Monte Carlo (MCMC) simulations to date the divergence of MHC IIB lineages that were found in at least four different genera of corvids, indicating that these are very old lineages, predating speciation by many millions of years. We propose that these lineage divergences represent MHC locus duplications of the single *DAB*1 locus that has been proposed as the progenitor of all MHC IIB loci in the Passeriformes [31]. Our calculations indicate that this duplication occurred just prior to the passerine radiations, near the time of the split between the passerine and parrot avian lineages. We propose that extant forms of these ancient lineages represent groups of paralogous loci that continue to be duplicated, neofunctionalized and often deleted in a manner consistent with the birth and death/accordion models of MHC evolution.

## Materials and Methods

### Sample collection, library preparation and sequencing

The collection and processing of the crow samples (n = 18 individuals of each species) are described elsewhere [22]. gDNA for the other corvid species (N = 4 for *P*. *pica*, *G*. *glandarius*; N = 3 *C*. *Cyanus*, *C*. *frugilegus*) was obtained from the Japanese National Museum of Nature and Science (Tsukuba, Japan) and the Yamashina Institute of Ornithology (Chiba, Japan). These samples were collected between 1994 and 2010 from the Japanese archipelago and Korea. Sample locations and dates for each species are listed in S1 Table.

Library preparation was carried out according to the methods described in [22]. Briefly, Fusion primers were synthesized (Fasmac, Japan) according to the Roche “Basic Amplicon” design by ligating the standard Roche multiplex identifiers (MIDs) to both the forward and reverse MHC IIB primers and the 454 adaptor primers. This method ensures that reads are sorted by unique MIDs on both ends of each amplicon which reduces the possibility of “tag switching”, which can lead to variant misidentification [32, 33]. These MHC IIB primers, *ComaiF2* and *ComaEx2RA*, were developed during our previous study and amplified a 248 bp fragment (excluding primers) of MHC IIB exon 2 in three species of *Corvus* (6). PCRs contained 25–50 ng of template, 0.5 μM of each fusion primer, 0.5 mM of each dNTP, 1.5mM MgCl_2_, 1X PCR buffer, 5% DMSO and 0.5 U of ExTaq (Takara, USA) polymerase (25 μL total volume). The cycling conditions included an initial denaturation at 94 ^◦^C for 30 s followed by 28 cycles of 94 (20 s), 60 (20 s) and 72 (45 s) ^◦^C. Samples were purified, quantified and quality checked according to the methods described in [22].

The amplicons were pooled and sequenced using the Roche GS Junior Titanium Sequencing System at the Japanese National Museum of Nature and Science in Tsukuba, Japan.

Amplicons were bi-directionally sequenced, and reads that passed the initial 454 Roche Junior quality filter were de-multiplexed using jMHC [34]. The sequences used in this study are the products of two independent 454 runs. The first run, described in [22], contained only MHC IIB amplicons from jungle crows, America crows and carrion crows. The second run contained a MHC IA library of crows (for a separate study) with the addition of the IIB amplicons from the corvid species described above. Because the amplicons (MHC IA and IIB) were nearly identical in size (248 bp versus 246), it is highly unlikely that there was read bias due to preferential 454 amplification of smaller reads. De-multiplexed FASTA files (excluding primers and MIDs) were then imported into Geneious v. 6.1 (Biomatters, NZ) and aligned with published *Corvus* class IIB sequences. Sequences less than 270 bp in length or containing indels were removed from the data set at this time.

MHC IIB variant validation and individual genotyping was carried out using a modified protocol of Galan et al. (2010) [35] as described previously [22]. Briefly, variants were validated if at least three identical reads were confirmed by two independent PCRs of the same individual. Independent PCR greatly reduces the inclusion of PCR generated chimeras, as the probability of amplifying the exact chimera twice is highly unlikely. To reduce the probability of erroneously including single base substitutions, we classified sequences as true variants only when they differed by at least three nucleotide substitutions from more common variants.

### Selection, phylogenetics and genetic lineage dating

Unique, validated MHC IIB variants were pooled for each species and aligned (both nucleotide and amino acid sequences) using Genious v. 6.1. Codons previously identified belonging to the PBR in humans [36] were identified and partitioned. We tested for selection by completing a d_N/_d_S_ ratio based test for selection (Codon Z Test; modified Nei-Gojobori with Jukes-Cantor correction) in MEGA v. 6.0 [37]. In order to construct a phylogeny, we first performed a best fit model test in MEGA. A Kimura 2-parameter (K2) model had the lowest BIC scores and a maximum likelihood (ML) tree was constructed in MEGA using a 248 bp MHC IIB fragment comparing all nucleotide substitutions with 500 bootstrap replicates. Rather than partitioning the fragments, we chose to use the entire 248 bp fragment because, although trees constructed from synonymous sites are thought to better reflect genetic ancestry (TSP) than those that include non-synonymous sites, [15] the low synonymous substitution rate typically found in coding genes requires fragments much larger than 248 bp in order to have enough statistical power to construct supported trees. While it has been shown that analyses using intron 2, rather than exon 2, provide less distorted evolutionary histories of MHC [20, 38], robust phylogenetic analyses, including investigations of TSP, using exon 2 in passerines have been successfully performed [23].

### Allelic lineage dating

In order to estimate the time of gene divergence, we calculated the time to the most recent common ancestor (MRCA) for each group of variants that clustered on the tree with at least 75% bootstrap support in the ML tree. In order to focus on those lineages that are likely to be ancient ones within the Corvidae, we limited these lineages to those that included representatives from all four corvid genera. We tested for nucleotide saturation using DAMBE [39]. The MRCA for each lineage was estimated in BEAST v. 2.0 using a HKY site model, with a Gamma setting of 4 and estimated Gamma shape value. A Yule process speciation prior for branching rates was used [40]. In order to account for heterogeneity of evolutionary rates within and between allelic lineages, we completed the simulations using two different clock settings: a relaxed clock-exponential setting and a random local clock [41]. In addition, we used the above methods to estimate the divergence time of each lineage from each other as well as the divergence time estimate for all lineages together. In order to reconstruct the probable evolutionary relationships of the lineages to each other, we reconstructed parsimony ancestral relationships with a Jukes Cantor model in Mesquite v.3.04 [42].

### Substitution rates for MHC IIB lineages

The use of previously estimated neutral nuclear gene mutation rates was inappropriate to estimate divergence of these allelic lineages because these genes are assumed to be under functional constraint, and thus, they are not evolving neutrally [43]. Therefore, we accounted for the functional constraint for each gene lineage by calculating an “adjusted mutation rate” in the following way: The synonymous substitution rate was plotted against the total substitution rate for all possible sequence pairs in a specific, supported gene lineage and the slope of this line (the estimate of functional constraint) was then multiplied by the previously estimated neutral nuclear gene mutation rates calculated for zebra finch (*Taeniopygia guttata*): 2.21 × 10^−9^ site^-1^ year^-1^ and domestic chicken (*Gallus gallus*): 1.91 × 10^−9^ site^-1^ year^-1^ [43, 44]. In this manner, we calculated estimated substitution rates for input into BEAST v. 2.0 for each gene lineage. For the divergence of lineage pairs, we used the average substitution rate of all four lineages. Next, Markov chain Monte Carlo (MCMC) analyses were run for 10^7^ generations (10,000 iteration burn in); the mean and 95% highest posterior density interval (HPD) for divergence times and other parameters were calculated in Tracer v. 1.6 [40]. While we report the results for using both zebra finch and chicken adjusted mutation rates, we highlight results using the passerine zebra finch estimate. While this rate is relatively high compared to other vertebrates, it was calculated using a whole genome approach, and is therefore, to our knowledge, the most comprehensive neutral mutation rate estimate for a passerine species [44]. We note that the generation time of zebra finches (*ca*. 3 months– 1 year [45] is considerably less than the corvid birds used in this study (*ca*. 1–3 years, [46, 47]), and therefore may underestimate divergence times [48]; however, the corvids used in this study are not physiologically constrained to long generation times, but rather social adaptations induce delayed breeding in some, but not all corvid species, and thus longer generation may be a recently derived trait [49]. In addition, there is no way to know if current passerine mutation rates are reflective of ancestral rates throughout the passerine radiation. Therefore, there is no “good” estimate for the mutation rate throughout the passerine radiations, and so we report results using both passerine and chicken estimated rates.

## Results

### Pyrosequencing

From the first sequencing run (fully described in [22]), a total of 107,279 reads passed the initial 454 filter. After excluding reads of less than 270 bp, there were 90,203 reads with an average length of 340 nucleotides. Individuals had between 275–1121 reads. From the second sequencing run, a total of 98,569 reads passed the initial filter, and after excluding reads less than 270 bp, there was a total of 84,254 reads with an average length of 349 nucleotides. We extracted the corvid MHC IIB sequences from the rest of the data set and calculated an average of 189–1544 reads per individual. GenBank Accession numbers for all sequences used in the study are: KP888319—KP888555; KU851041—KU851133.

### Genetic variation, test for selection and phylogenetic reconstruction

Because the MHC IIB sequence variation was over represented in the three crow species (N = 18 birds) relative to the other species (N = ~ 4 birds), we selected a subset of sequences from these three crow species to use in subsequent analyses. This was necessary in order to generate a tree of manageable size for visualization. This was done by constructing an ML tree using all of the crow sequences and then removing crow variants from supported clusters that were over-represented. We retained up to six sequences from each crow species in the four supported intergenic lineages.

For the species from the second sequencing run, after trimming the sequences and removing those with indels and stop codons, an alignment with previously published *Corvus* MHC IIB exon 2 sequences showed that the primer pair amplified the same MHC IIB region as in crows [22]. Table 1 provides the relevant genetic information for each species genotyped including average number of variants/species, overall nucleotide variation, synonymous and non-synonymous substitution rates (d_S_ and d_N_, respectively), d_N_ / d_S_ and the results of the Z-test for selection using both the entire fragment and the PBR partition and Tajima’s D. All seven species exhibited similar nucleotide variation (range: 0.160–0.188) across the 248 bp fragment. While there was substantial variation of d_N_ and d_S_ between species, in all cases the d_N_ / d_S_ were similar (range: 1.3–1.6) and positive selection in all species was detected using both the entire fragment and the PBR partition (Table 1).

**Table 1:** Statistics for 248 bp fragment of MHC IIB exon 2 in corvid birds used in this study. *Cobr* = American crow; *Coco* = carrion crow; *Coma* = jungle crow; *Cofr* = Asian rook; *Gagl* = Eurasian jay; *Pipi* = Eurasian magpie; *Cycy* = azure-winged magpie.

Species	*n*	Variants (total)	Variants (individual)	*π*^1^	*π* d_S_^2^	*π* d_N_^3^	d_N_ / d_S_	*P*-value Z-test^4^	*P*-value Z-test PBR^5^
*Cobr*	18	33	11–18	0.162	0.119	0.177	1.5	<0.0001	0.008
*Coco*	18	56	12–20	0.160	0.113	0.179	1.6	0.001	0.005
*Coma*	18	31	7–18	0.168	0.134	0.181	1.3	0.004	0.01
*Cofr*	3	27	8–15	0.179	0.136	0.194	1.4	0.001	0.006
*Gagl*	4	28	9–19	0.177	0.147	0.188	1.3	0.002	0.0015
*Pipi*	4	20	9–11	0.175	0.128	0.194	1.5	<0.0001	0.003
*Cycy*	3	18	8–12	0.188	0.151	0.201	1.3	0.001	0.008

^1^ nucleotide diversity

^2^ synonymous substitution rate

^3^non-synonymous substitution rate

^4^codon test for selection using entire fragment

^5^codon test for selection using the peptide binding region partition from Brown *et al*. [36]

**Fig 1:**
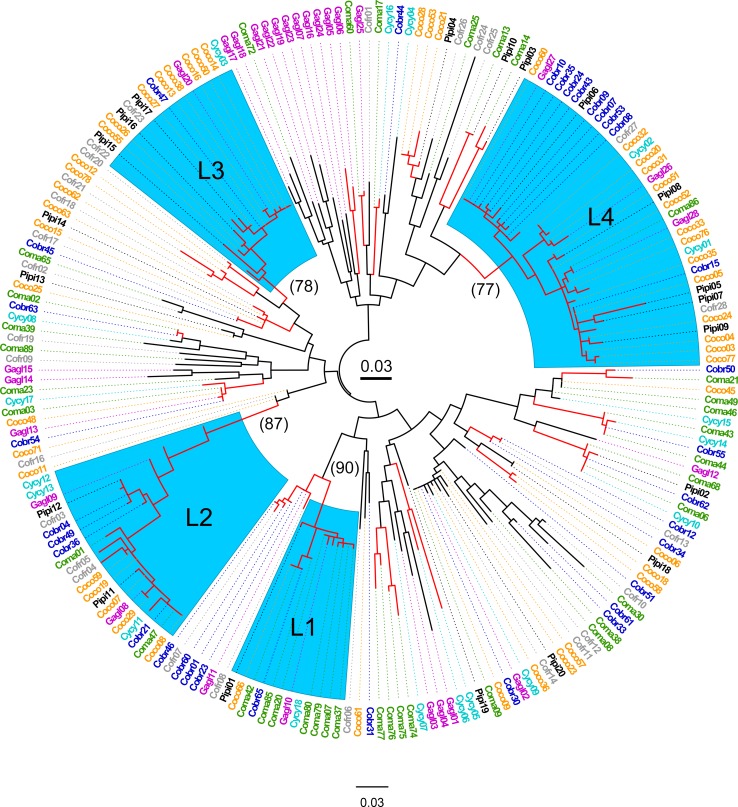
Maximum likelihood tree of MHC IIB variants (248 bp) in seven species of Corvidae. Four lineages (L1-L4) that contained four different genera clustered with > 75% bootstrap support (blue highlight). Pertinent bootstrap values are in parentheses. Cobr = American crow; Coco = carrion crow; Coma = jungle crow; Cofr = Asian rook; Gagl = Eurasian jay; Pipi = Eurasian magpie; Cycy = Azure-winged magpie. Red branches indicate > 70% bootstrap support. Scale bar in center indicates substitutions/site.

**Fig 2:**
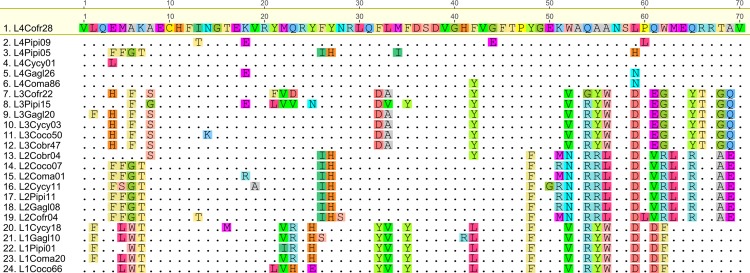
Amino acid alignment of representative sequences from each of six supported MHC IIB lineages. Each lineage (L1 –L4) contains four genera. Dots indicate amino acids identical to reference (L4Cofr28). Cobr = American crow; Coco = carrion crow; Coma = jungle crow; Cofr = Asian rook; Gagl = Eurasian jay; Pipi = Eurasian magpie; Cycy = Azure-winged magpie.

The ML tree revealed four well supported clades (>75% bootstrap support) with each containing all four genera, and one lineage with three genera (Fig 1). Further evidence of TSP was shown in the amino acid alignments of the four major allelic lineages which showed striking motif sharing within lineages, and even in some cases, identical amino acid sequences shared among genera (Fig 2). While it was not possible to assign alleles to specific loci, the four allelic lineages identified share distinct nucleotide and amino acid motifs, and group into well supported clades, suggesting that these lineages belong to either individual loci, or, more likely, to distinct groups of paralogous loci (Figs 1 and 2).

### Age of allelic lineages

The test for saturation in DAMBE revealed little saturation effect: Iss < Iss.c. (*P* < 0.0000). The adjusted mutation rates for each lineage using the zebra finch rate were calculated at: L1 = 0.00191, L2 = 0.00162, L3 = 0.00182, L4 = 0.0014 (per site/million years). Using the chicken adjusted rate these values were L1 = 0.00212, L2 = 0.00148, L3 = 0.00165, L4 = 0.00132. The average adjusted rate (across all four lineages) was 0.00160 using the zebra finch rate and 0.00177 using the chicken rate.

### MCMC simulations using the zebra finch adjusted mutation rate

The estimate of the mean posterior distribution to the MRCA was similar whether using the random local clock or the relaxed log normal clock; however, the HPDs using the random local clock had considerably lower ranges than those calculated from the relaxed clock. Summary statistics for both clock models are reported in S2 and S3 Tables. When comparing the HPDs, the mean range when using the random local clock was 27.7 my while the mean range for the relaxed clock was 37.7 my, a difference of ~27%. In addition, the standard errors of the mean as well as the standard deviations were less using the random clock and BEAST output estimated sample size (ESS) values for all statistical categories were > 100. Thus, the following results are reported using the random local clock setting.

**Fig 3:**
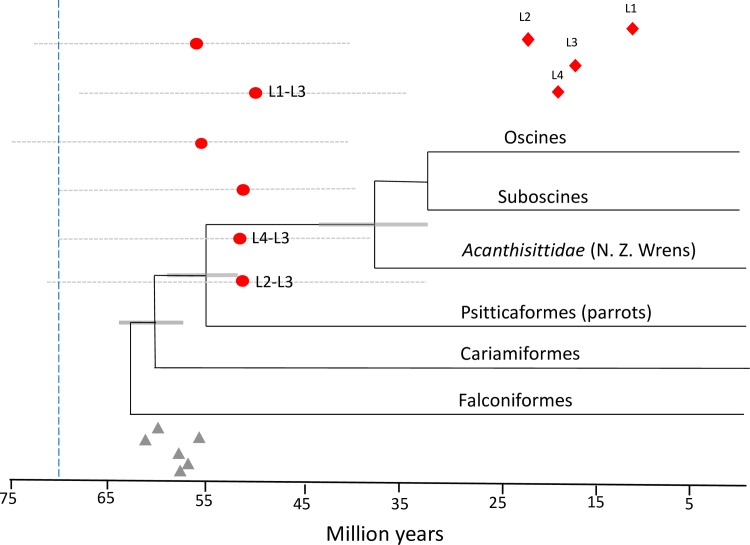
MCMC simulations performed in BEAST v. 2.0 using the zebra finch adjusted mutation rate. Mean estimates of divergence between each pair of lineages (red circles) and the associated 95% highest posterior density interval (HPD) for each posterior distribution (gray dotted lines). Lineage divergences proposed by simulated ancestral states are labeled: L1-L3, L4-L3, L2-L3. Red diamonds = mean divergence time in millions of years (ma) of four supported MHC IIB lineages (L1-L4) in the Corvidae. Gray triangles are the mean estimates of the MRCA for the all lineage dyads using the adjusted chicken mutation rate. The phylogenetic reconstruction and estimated divergence times of major clades are from Jarvis et al. (2014). Blue vertical dashed line indicates an alternate estimated divergence time of suboscine and oscine clades (Ericson et al. 2014).

**Fig 4:**
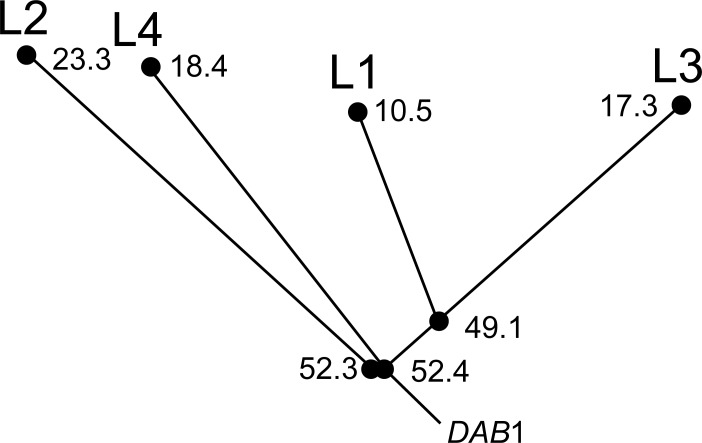
Proposed evolutionary history of the major MHC IIB lineages in corvids. The cladogram was constructed from the ancestral state reconstruction conducted in Mesquite combined with the ML tree and MCMC divergence time data. Mean divergence times are indicated next to major nodes. Inclusion of *DAB*1 as the progenitor locus is based on evidence provided by Burri et al. (2008) [20].

The time to the MRCA for each of the four allelic lineages ranged from a mean of 10.0–23.3 ma. Divergence time estimates for all lineages and lineage-lineage divergences are found in Fig 3. Summary statistics, including MRCA (mean posterior distribution), HPDs, standard errors of the means and standard deviations are reported in S2 Table. By combining the ancestral reconstructions from the Mesquite analysis, the ML tree and the estimated divergence times, a picture of the evolutionary history of these lineages emerges as such: The L2, L3 and L4 lineages diverged from each other *ca*. 53 ma., while the L1 lineage diverged from the L3 lineage *ca*. 49.1 ma (Fig 4; S1 Fig). The estimated time to the MRCA for all lineages was 61.5 ma (HPD: [46.9; 76.4]).

As expected, the results of MCMC simulations using the chicken adjusted mutation rate revealed slightly older divergence times across all lineage comparisons. The same pattern of smaller HPD ranges and as well as standard errors and standard deviations when employing a random local clock versus a relaxed clock were also observed in these simulations. Summary statistics for divergence time using the chicken adjusted rate are shown in S3 Table and mean divergence times of lineages from each other are shown in Fig 3.

## Discussion

In this study we have provided evidence for the maintenance of ancient MHC IIB lineages among several genera of corvid birds, and to our knowledge, the strongest evidence for TSP in passerines to date. In addition, using phylogenetic and ancestral state reconstruction in combination with MCMC simulations we show that these MHC lineages likely represent the descendants of a single MHC IIB locus, which was previously shown by Burri et al. (2008) to be *DAB*1 [20].We suggest that this ancestral locus was duplicated multiple times at or near the time of the split between the Passeriformes (which appear to share the characteristic of highly duplicated MHC IIB) from the rest of the avian orders (which, except in rare cases, exhibit two–three MHC IIB loci [14]). Furthermore, sample size limitations mean that we likely did not uncover all of the MHC IIB lineages present within the Corvidae, as several intergenic clusters were observed, but not supported in the tree. The inclusion of additional corvid genera and increased sample sizes within species may resolve more allelic lineages within this family, with the implication that the *DAB*1 locus has experienced more than three duplications just prior to the major passerine radiations than the data presented here suggest.

Our data suggest an early duplication of the MHC in passerines, which is consistent with a recent analysis of the MHC IIB in Australian basal songbird lineages that showed supported clustering of MHC IIB alleles (TSP) among different families [25]. There are, however, two potentially contentious issues in this study that we would like to address; firstly, the effects of gene conversion on population genetic statistics and the phylogenetic signal of the MHC, and secondly, the avian divergence times that we used to relate our findings.

### Gene conversion and MHC

Gene conversion is known to affect the both the pattern of nucleotide substitutions and divergence estimates. Teshima and Innan (2004) assessed the effect gene conversion had on divergence time estimates on a system consisting of duplicated genes (such as the MHC). Using simulations, the authors determined that analyzing duplicated genes based on the molecular clock may be misleading if gene conversion is common [50]. Similarly, in an empirical study on gene conversion (recombination) in plant resistance genes (R genes), Jouet et al. (2015) found that this evolutionary force significantly altered the divergence time estimates of taxa, as well as the overall gene tree topology. Increased levels of polymorphism in recombinant regions resulted in a 14.3% overestimation of the age of the taxa in the phylogenetic tree using the total alignment compared to the tree with recombinant regions removed [28]. Given that gene conversion can result in concerted evolution, i.e. in the homogenization of MHC alleles within species, loci may appear less diverged from one another. This mechanism has been proposed to explain patterns observed in MHC phylogenies of songbirds, which often cluster by species rather than by locus (as in mammals etc. e.g. [27, 38, 51]). However, the MHC lineages used in our analysis showed a remarkable amino acid motif similarity between species, which we interpret as evidence against a strongly homogenizing force such as gene conversion. Indeed, it is difficult to envisage how this pattern could have been maintained over many millions of years under constant gene conversion across MHC loci. Nevertheless, in order to minimize the effect of gene conversion, we based our estimates on a random local clock, rather than a strict clock, and used calibration points which have been proposed to minimize the bias in age estimates introduced by recombination [28].

A further complication of gene conversion is its potential effect on substitution patterns and signals of selection. New MHC alleles are thought to be generated through both intra and inter-locus gene conversion, and while gene conversion does not increase overall MHC nucleotide diversity, it does increase functional allelic diversity [29]. Short-block gene conversion has been suggested to contribute to MHC diversity and increase the d_N/_d_S_ ratios, potentially leading to spurious signals of positive selection [38]. On the other hand, Spurgin et al. (2011) showed that short-block gene conversion (which they called “micro-recombination”) increased both d_N_ and d_S_, but that it had little impact on the d_N/_d_S_ ratios. Their study of Berthelot’s pipit (*Anthus berthelotii*), showed significantly elevated synonymous and non-synonymous substitutions in the peptide binding region of the MHC, and of the 29 sequences that evolved in the last 75,000 years, 27 gene conversion events and two point mutations could potentially explain all the MHC sequence variation in the pipit populations [52]. It is unclear how this pipit MHC system relates to the corvid species in our study; however, it is likely that gene conversion continues to generate allelic diversity in the species examined here. Indeed, most of the MHC IIB sequences in Fig 1 do not group into supported clades, and thus the loci these alleles are associated with may indeed experience higher rates of recombination than the established lineages (L1-L4). Our results and conclusions, however, rely on the sequence similarities found within the supported lineages, and it is unlikely that these similarities resulted from independent gene conversion events within each species.

### Avian divergence times

Our conclusion that multiple duplication events of a single MHC IIB locus occurred just prior to or during the split between the passerines and parrots is based on the avian divergence times estimated by Jarvis et al. (2014) (Fig 3). This analysis combined multiple fossil calibrations and genome wide nucleotide data sets to construct a highly resolved total evidence nucleotide tree (TENT). While the Jarvis et al. (2014) avian tree is by far the most comprehensive and arguably the most accurate estimate of divergence times within the avian class, we do not discount another recent estimate for the divergence times of the Passeriformes carried out by Ericson et al. (2014) [30]. This estimate argues for an older suboscine/oscine split within the Passeriformes (*ca*. 71 ma); although it was based on only nine nuclear genes and fewer fossil calibrations than the Jarvis et al. (2014) estimate. If the suboscine/oscine split is indeed much older than generally accepted (*ca*. 71 vs. *ca*. 31 ma), our results indicate that the MHC IIB duplication event occurred after the suboscine/oscine split, near the beginning of the major songbird radiations (Fig 3). This is conceivable, and the possibility could be resolved by analysis of suboscine MHC IIB systems; however, no studies to date have provided any estimates of the number of MHC IIB loci found in extant suboscines.

### Evolutionary success of passerines

The reasons for the unparalleled success of songbirds, in terms of species diversification and range expansion, remain to be answered; however, a few key adaptations have been proposed. These passerine-specific adaptations, or synapomorphies, include spermatozoa and palate morphology, relatively larger brains and higher cognitive ability compared to other birds, increased vocalization skills and generally small body size [10, 53, 54]. In this study, the divergence time of three of the four corvid MHC IIB allelic lineages was *ca*. 53 ma, which corresponds to the divergence of the passerine clade from the parrot clade [13]. We suggest that the similar timing of these ancient duplications and the divergence of passerines from other avian groups may not be coincidental, but rather may be related to the success of this clade.

These first duplications have likely been followed by further duplications (and deletions) that explain the high number of MHC loci observed in extant passerines. Highly duplicated MHC genes, whether functional or not, could serve as reservoirs for pathogen-resistance associated genetic polymorphisms [1, 29] that enabled the passerines to radiate faster and wider than any other avian group, and thus should be considered among the possible synapomorphies that explain their unrivaled taxonomic and ecological diversity among terrestrial vertebrates.

While research on adaptive evolution and speciation theory has historically focused on the MHC, there are many other multigene families in the immune genome of birds that show high levels of polymorphism and strong signatures of positive selection, perhaps most notably the avian β-defensins (AvBDs) and Toll-like receptor (TLRs) [55, 56]. Further analyses of these immune gene families and comparisons with the MHC are warranted to examine whether the unique expansion of the MHC has indeed been instrumental in the evolutionary success of passerines.

## Conclusions

The results presented here provide compelling evidence that the highly duplicated MHC IIB observed in passerine birds is primarily the result of ancient gene duplication, although more recent duplication and recombination likely continue to shape the MHC IIB of songbirds. Estimates of divergence between different corvid lineages suggest that a major MHC IIB duplication event coincides with the divergence of the Passeriformes from other avian orders *ca*. 53 ma. Since the MHC is a critical component of pathogen recognition and immune response, the expansion of this gene complex in passerines may have facilitated more rapid range expansion and increased species survival relative to other avian orders. We argue that duplication of the MHC early in the passerine radiations is a potential synapomorphy that contributed to the unparalleled success of this order.

## Supporting Information

S1 Fig Ancestral state reconstruction of each MHC IIB lineage found among four corvid genera using parsimony jukes cantor analysis in Mesquite. Included is a cladogram showing the bootstrap values (500 replicates) from a ML reconstruction using the K-2 model.
(DOCX)

S1 Table Collection sites and dates for all samples in this study. (DOCX)

S2 Table Comparison of MCMC clock settings using the adjusted mutation rate of zebra finch in BEAST v.2.0. (DOCX)

S3 Table Comparison of MCMC clock settings using the adjusted mutation rate of chicken in BEAST v.2.0. (DOCX)

S4 Table Specimen identification numbers for samples from the Japanese National Museum of Nature and Science and from the Yamashina Institute of Ornithology. (DOCX)
