# Supplementary material for: Early Duplication of a Single MHC IIB Locus Prior to the Passerine Radiations
Source: PLoS One. 2016 Sep 22;11(9):e0163456. doi: 10.1371/journal.pone.0163456 (PMC5033386; doi:10.1371/journal.pone.0163456)

S1 Fig. **Ancestral state reconstruction of each MHC IIB lineage found among four corvid genera using parsimony jukes cantor analysis in Mesquite.** Included is a cladogram showing the bootstrap values (500 replicates) from a ML reconstruction using the K-2 model.


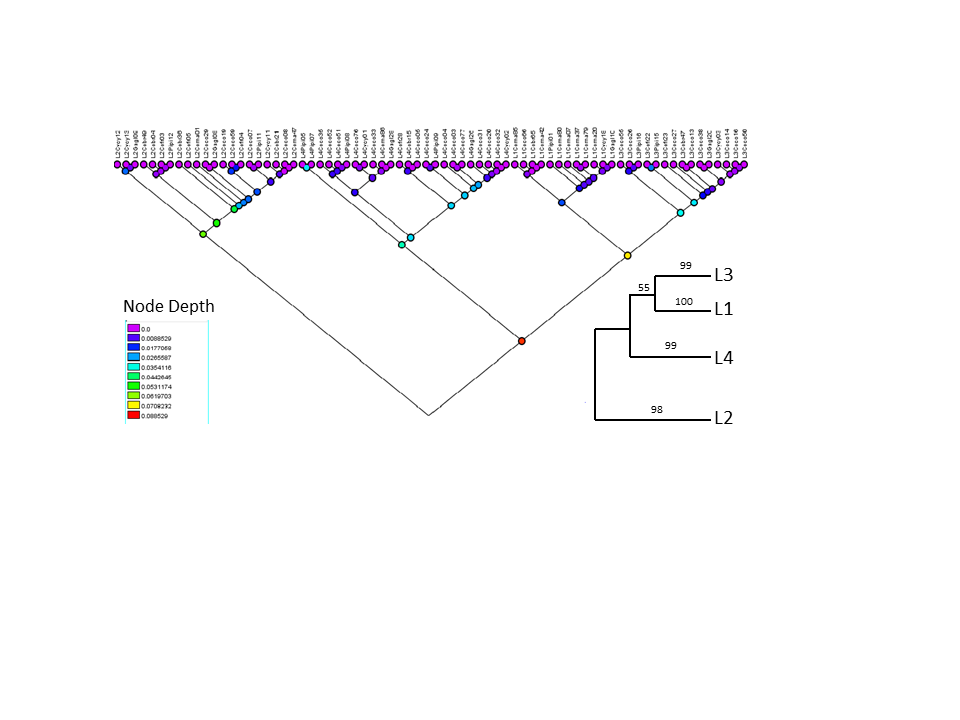

Supplement: S1 Fig — Included is a cladogram showing the bootstrap values (500 replicates) from a ML reconstruction using the K-2 model. (DOCX) [file pone.0163456.s001.docx]
