# Supplementary material for: Early Duplication of a Single MHC IIB Locus Prior to the Passerine Radiations
Source: PLoS One. 2016 Sep 22;11(9):e0163456. doi: 10.1371/journal.pone.0163456 (PMC5033386; doi:10.1371/journal.pone.0163456)
Supplement: S1 Table — (DOCX) [file pone.0163456.s002.docx]

**S1 Table.** **Collection sites and dates for all samples in this study.**

| **Species** | **Locaton** | **Date** |
| --- | --- | --- |
| American crow | UC-Davis, CA USA (18) | 2012 |
| jungle crow | Tokyo, Japan (16) | 1994-2010 |
|  | Hokkaido, Japan (1) | 2007 |
|  | Okinawa, Japan (1) | 2008 |
| carrion crow | Kagoshima, Japan (6) | 2002-2004 |
|  | Tsushima, Japan (1) | 2003 |
|  | Nara, Japan (4) | 2003 |
|  | Osaka, Japan (1) | 2007 |
|  | Tokyo, Japan (6) | 2008 |
| Asian rook | Akita, Japan (4) | 2006 |
| Eurasian jay | Miyagi, Japan (1) | 2004 |
|  | Nagano, Japan (2)) | 2004 |
|  | Jeju-Do, Korea (1) | 2003 |
| Eurasian magpie | Daejon, Korea (4) | 2002 |
| Azure-winged magpie | Gangwon, Korea (1) | 2003 |
|  | Saitama, Japan (1) | 2004 |
|  | Tokyo, Japan (1) | 2007 |
