# Supplementary material for: Early Duplication of a Single MHC IIB Locus Prior to the Passerine Radiations
Source: PLoS One. 2016 Sep 22;11(9):e0163456. doi: 10.1371/journal.pone.0163456 (PMC5033386; doi:10.1371/journal.pone.0163456)
Supplement: S2 Table — (DOCX) [file pone.0163456.s003.docx]

**S2 Table. Comparison of MCMC clock settings using the adjusted mutation rate of zebra finch in BEAST v.2.0.**

| Random local clock | | | | | Relaxed clock log normal | | | |
| --- | --- | --- | --- | --- | --- | --- | --- | --- |
|  | Mean^a^ div | 95% HPD^b^ | SEM^c^ | Std Dev^d^ | Mean div | 95% HPD | SEM | Std Dev |
| L1 | 10.04 | 4.81-15.7 | 0.0313 | 2.89 | 10.2 | 4.75-16.5 | 0.0345 | 3.13 |
| L2 | 23.3 | 14.7-32.5 | 0.06 | 4.72 | 26.6 | 10.2-49.3 | 0.303 | 11.6 |
| L3 | 17.3 | 8.80-27.1 | 0.132 | 4.93 | 18.2 | 6.4-33.1 | 0.162 | 8.21 |
| L4 | 18.4 | 10.9-26.5 | 0.055 | 4.17 | 19.1 | 8.95-31.7 | 0.148 | 6.7 |
| L1L2 | 55.7 | 38.9-73.3 | 0.108 | 8.93 | 38.03 | 18.8-61.3 | 0.416 | 11.9 |
| L1L3 | 49.1 | 32.8-66.6 | 0.127 | 8.96 | 36.1 | 17.4-57.8 | 0.38 | 12.1 |
| L1L4 | 56.1 | 37.6-74.6 | 0.257 | 9.44 | 36.2 | 19.1-55.6 | 0.36 | 10.4 |
| L2L3 | 52.3 | 37.1-70.6 | 0.191 | 8.78 | 47.9 | 19.2-87.5 | 1.01 | 20.2 |
| L2L4 | 51.8 | 36.2-69.4 | 0.177 | 8.66 | 41.1 | 20.05-66.0 | 0.633 | 13.01 |
| L3L4 | 52.4 | 31.9-72.5 | 0.888 | 10.5 | 36.7 | 17.9-60.8 | 0.586 | 12.5 |
| ALL | 61.5 | 46.9-76.4 | 0.163 | 7.66 | 51.2 | 29.8-74.7 | 0.683 | 12.2 |

^a^ mean posterior distribution of MRCA

^b^ highest posterior density

^c^ standard error of the mean

^d^ standard deviation
