# Supplementary material for: Early Duplication of a Single MHC IIB Locus Prior to the Passerine Radiations
Source: PLoS One. 2016 Sep 22;11(9):e0163456. doi: 10.1371/journal.pone.0163456 (PMC5033386; doi:10.1371/journal.pone.0163456)
Supplement: S3 Table — (DOCX) [file pone.0163456.s004.docx]

**S3 Table. Comparison of MCMC clock settings using the adjusted mutation rate of chicken in BEAST v.2.0.**

| RANDOM LOCAL CLOCK | | | | | RELAXED CLOCK LOG NORMAL | | | |
| --- | --- | --- | --- | --- | --- | --- | --- | --- |
|  | Mean div^a^ | 95% HPD^b^ | SEM^c^ | Std Dev^d^ | Mean div | 95% HPD | SEM | Std Dev |
| L1 | 9.244 | 4.6-14.5 | 0.029 | 2.64 | 9.28 | 4.5-14.9 | 0.03 | 2.75 |
| L2 | 28.2 | 17.6-39.5 | 0.074 | 5.73 | 31.8 | 11.8-59.7 | 0.394 | 15.4 |
| L3 | 18.9 | 9.4-29.5 | 0.15 | 5.44 | 18.8 | 6.5-34.7 | 0.188 | 8.81 |
| L4 | 24.7 | 14.5-36.01 | 0.095 | 5.68 | 24.4 | 11.7-40.1 | 0.166 | 8.23 |
| L1L2 | 60.9 | 42.8-81.1 | 0.157 | 9.82 | 41.7 | 19.9-67.0 | 0.501 | 13.2 |
| L1L3 | 55.8 | 37.1-76.2 | 0.143 | 10.2 | 38.7 | 19.0-63.0 | 0.344 | 12.1 |
| L1L4 | 62.2 | 43.4-82.9 | 0.234 | 10.3 | 38.4 | 21.0-59.9 | 0.371 | 10.5 |
| L2L3 | 57.7 | 39.5-77.5 | 0.263 | 9.86 | 50.5 | 21.5-90.1 | 1.31 | 21.8 |
| L2L4 | 57.4 | 39.0-76.1 | 0.196 | 9.57 | 44.9 | 21.1-72.5 | 0.664 | 14.5 |
| L3L4 | 58.4 | 37.5-80.7 | 0.5404 | 11.2 | 40.8 | 20.0-60.7 | 0.575 | 13.6 |
| ALL | 68.01 | 52.1-85.1 | 0.164 | 8.51 | 54.9 | 31.2-82.4 | 0.921 | 13.6 |

^a^ mean posterior distribution of MRCA

^b^ highest posterior density

^c^ standard error of the mean

^d^ standard deviation
