# Supplementary material for: Early Duplication of a Single MHC IIB Locus Prior to the Passerine Radiations
Source: PLoS One. 2016 Sep 22;11(9):e0163456. doi: 10.1371/journal.pone.0163456 (PMC5033386; doi:10.1371/journal.pone.0163456)
Supplement: S4 Table — (DOCX) [file pone.0163456.s005.docx]

**S4 Table. Specimen identification numbers for samples from The Japanese National Museum of Nature and Science (JNMNS) and the Yamashina Institute of Ornithology (YIO).**

|  | Specimen | Location | species |  |
| --- | --- | --- | --- | --- |
|  | ID |  |  |  |
| 1 | B-002 | YIO | C. corone |  |
| 2 | B-003 | YIO | C. corone |  |
| 3 | B-006 | YIO | C. corone |  |
| 4 | B-007 | YIO | C. corone |  |
| 5 | B-008 | YIO | C. corone |  |
| 6 | B-009 | YIO | C. corone |  |
| 7 | B-010 | YIO | C. corone |  |
| 8 | B-020 | YIO | C. corone |  |
| 9 | B-022 | YIO | C. corone |  |
| 10 | B-023 | YIO | C. corone |  |
| 11 | B-024 | YIO | C. corone |  |
| 12 | B-025 | YIO | C. corone |  |
| 13 | B-026 | YIO | C. corone |  |
| 14 | B-040 | YIO | C. corone |  |
| 15 | CC7321-1 | JNMNS | C. corone |  |
| 16 | CC7316-1 | JNMNS | C. corone |  |
| 17 | CC7947-1 | JNMNS | C. corone |  |
| 18 | CC7946-1 | JNMNS | C. corone |  |
| 19 | 50061 | JNMNS | C. macrorhynchos | |
| 20 | 9521 | JNMNS | C. macrorhynchos | |
| 21 | 9523 | JNMNS | C. macrorhynchos | |
| 22 | 50525 | JNMNS | C. macrorhynchos | |
| 23 | 50524 | JNMNS | C. macrorhynchos | |
| 24 | 50613 | JNMNS | C. macrorhynchos | |
| 25 | 50615 | JNMNS | C. macrorhynchos | |
| 26 | 50618 | JNMNS | C. macrorhynchos | |
| 27 | 50619 | JNMNS | C. macrorhynchos | |
| 28 | 50625 | JNMNS | C. macrorhynchos | |
| 29 | 9644 | JNMNS | C. macrorhynchos | |
| 30 | 9790 | JNMNS | C. macrorhynchos | |
| 31 | 7740 | JNMNS | C. macrorhynchos | |
| 32 | 7741 | JNMNS | C. macrorhynchos | |
| 33 | 7743 | JNMNS | C. macrorhynchos | |
| 34 | 7744 | JNMNS | C. macrorhynchos | |
| 35 | 7745 | JNMNS | C. macrorhynchos | |
| 36 | 7746 | JNMNS | C. macrorhynchos | |
| 37 | 2589/52 | JNMNS | C. cyanus |  |
| 38 | 4036/133 | JNMNS | C. cyanus |  |
| 39 | 8627/48 | JNMNS | C. cyanus |  |
| 40 | 5870/186 | JNMNS | C. frugilegus | |
| 41 | 5873/189 | JNMNS | C. frugilegus | |
| 42 | 5874/190 | JNMNS | C. frugilegus | |
| 43 | 3496 | JNMNS | G. glandarius | |
| 44 | 3495 | JNMNS | G. glandarius | |
| 45 | 3494 | JNMNS | G. glandarius | |
| 46 | 3401 | JNMNS | G. glandarius | |
| 47 | 3309 | JNMNS | p. pica |  |
| 48 | 3310 | JNMNS | p. pica |  |
| 49 | 3311 | JNMNS | p. pica |  |
| 50 | 3312 | JNMNS | p. pica |  |
